# A subset of liver resident natural killer cells is expanded in hepatitis C-infected patients with better liver function

**DOI:** 10.1038/s41598-020-80819-8

**Published:** 2021-01-15

**Authors:** Erin H. Doyle, Costica Aloman, Ahmed El-Shamy, Francis Eng, Adeeb Rahman, Arielle L. Klepper, Brandy Haydel, Sander S. Florman, M. Isabel Fiel, Thomas Schiano, Andrea D. Branch

**Affiliations:** 1 Division of Liver Diseases, Icahn School of Medicine at Mount Sinai School, 1425 Madison Ave., Icahn 11-23, New York, NY 10029 USA; 2 Rush University Medical Center, Chicago, IL USA; 3 Human Immune Monitoring Core, Icahn School of Medicine at Mount Sinai, New York, NY USA; 4 Recanati Miller Transplantation Institute, The Mount Sinai Hospital, New York, NY USA; 5 Department of Pathology, The Mount Sinai Hospital, New York, NY USA

**Keywords:** Hepatology, Hepatitis, Viral hepatitis, Hepatitis C

## Abstract

Viral hepatitis leads to immune-mediated liver injury. The rate of disease progression varies between individuals. We aimed to phenotype immune cells associated with preservation of normal liver function during hepatitis C virus (HCV) infection. Clinical data and specimens were obtained from 19 HCV-infected patients undergoing liver transplantation. Liver and peripheral blood mononuclear cells were isolated and eight subsets of innate immune cells were delineated by multiparameter flow cytometry. Cytokine assays and microarrays were performed. Intrahepatic CD56^Bright^/CD16^-^ natural killer (NK) cells comprised the only subset correlating with better liver function, i.e., lower bilirubin (*p* = 0.0002) and lower model for end stage of liver disease scores (*p* = 0.03). The signature of liver NK cells from HCV-infected patients included genes expressed by NK cells in normal liver and by decidual NK cells. Portal vein blood had a higher concentration of interleukin (IL)-10 than peripheral blood (*p* = 0.03). LMCs were less responsive to toll-like receptor (TLR) stimulation than PBMCs, with fewer pro-inflammatory gene-expression pathways up-regulated after in vitro exposure to lipopolysaccharide and a TLR-7/8 agonist. Hepatic CD56^Bright^/CD16^-^ NK cells may be critical for maintaining liver homeostasis. Portal vein IL-10 may prime inhibitory pathways, attenuating TLR signaling and reducing responsiveness to pro-inflammatory stimuli.

## Introduction

The global burden of advanced liver disease is increasing rapidly, far out stripping increases in cardiovascular and chronic pulmonary diseases^1^. It is therefore a priority to increase knowledge of the hepatic cells, including the immune cell subsets, that preserve and restore liver function. Liver immune cells have distinctive functional properties. They cleanse portal vein blood of lipopolysaccharide (LPS) and other microbial toxins without undergoing pro-inflammatory responses^2^ and create a tolerogenic microenvironment that protects liver grafts from chronic rejection^3^. Single cell RNA sequencing and mass cytometry are revealing the heterogeneity of human liver immune cells^4,5^ and highlighting the need to integrate data about cell populations with clinical data so that translational research can focus on the key subtypes.

Approximately 1.34 million people die each year from hepatitis B virus (HBV) and hepatitis C virus (HCV) infection^6^. Direct acting antiviral drugs allow most HCV infections to be cured and reduce HBV disease; however, these viruses remain major public health threats. Patients cured of HCV continue to have an elevated risk of liver-related morbidity and mortality. Regardless of etiology, the extent of liver damage varies from person to person as a consequence of genetic, environmental, and immunological factors. Although liver damage is often immune-mediated^7^; immunosuppression can *accelerate* pathogenesis, as seen in HCV-infected patients with inborn immunodeficiencies^8^, HIV infection^9^, or on immunosuppressive drugs^10^, implying that, in the absence of immunosuppression, cytotoxic immune cells are held in check by other immune cell subsets. A promising strategy for improving outcomes in liver disease patients is to identify the immune cell subsets that modulate immunopathology, promote repair, and maintain homeostasis.

In this study, we analyzed innate immune cell populations in liver and peripheral blood of 19 HCV-infected adults undergoing liver transplantation, seeking subsets expanded in patients with relatively well-preserved liver function. Model for end stage liver disease (MELD) scores ranged from 7 to 42 in the study group. Uniquely, liver CD56^Bright^/CD16^-^ natural killer (NK) cells were expanded in patients with lower MELD scores and lower serum bilirubin, consistent with evidence that the abundance of intrahepatic NK cells correlates inversely with serum transaminase values and ISHAK fibrosis scores in HCV-infected patients^11,12^. Gene expression profiling showed a relationship between liver NK cells and decidual NK cells; these cells promote placental formation largely by secreting growth factors, rather than by killing neighboring cells^13^. If liver CD56^Bright^/CD16^-^ NK cells act through similar mechanisms, greater knowledge of their secretome and cell to cell interactions could lead to interventions that increase their activity and thereby improve liver function.

## Results

### NK cells dominate the intrahepatic innate immune cell population and transcriptome

**Figure 1:**
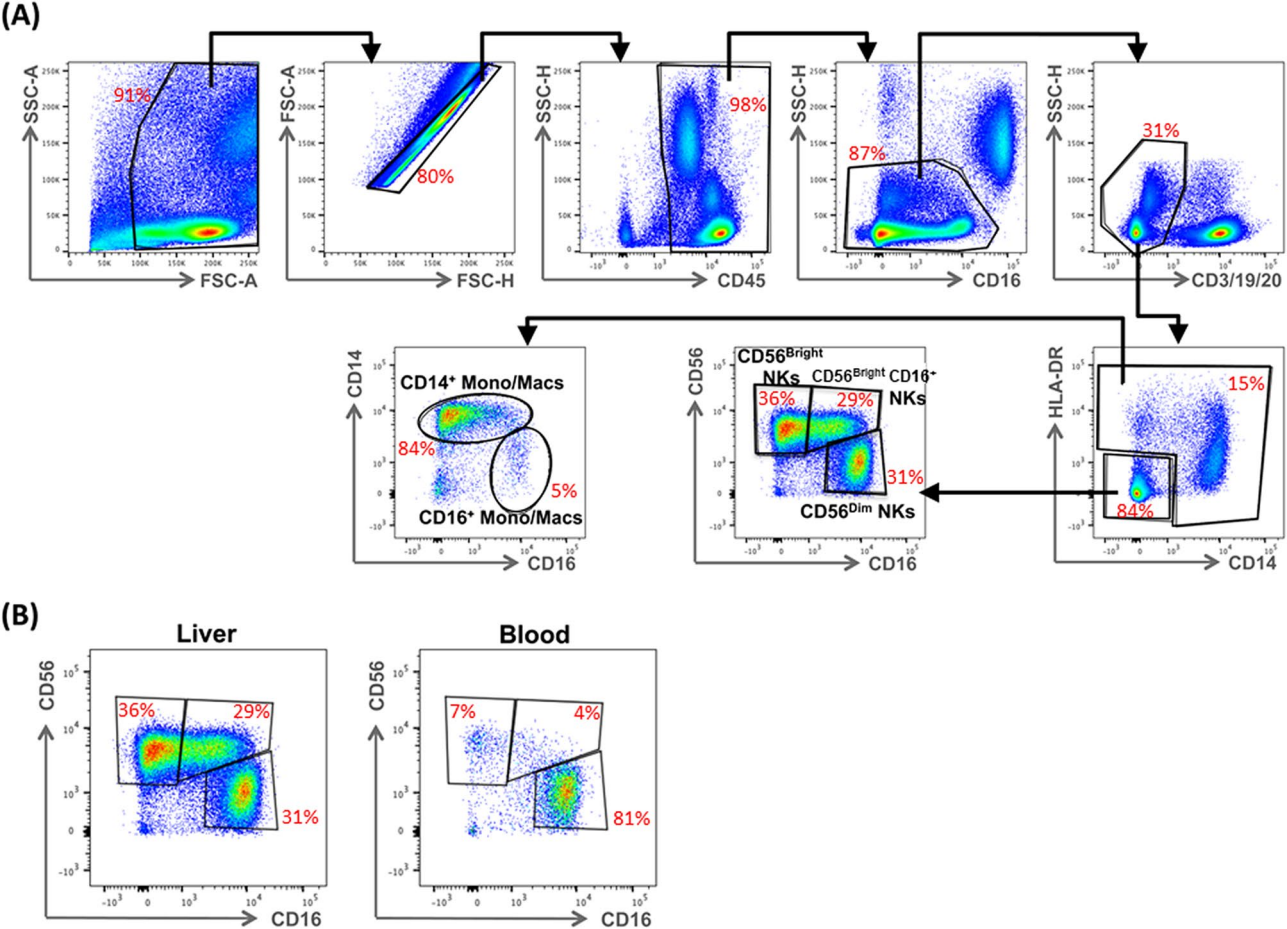
Identification of hepatic innate immune cell populations by multiparameter flow cytometry, the gating strategy. **(A)** Hepatic mononuclear cells from a representative liver were stained with a nine-color antibody panel: CD45, CD3, CD19, CD20, HLA-DR, CD14, CD16, CD123, BDCA1, BDCA3, and CD56. Innate immune mononuclear cells were selected based on viability (live/dead), unicellularity (singlets), CD45 expression (CD45^+^), intracellular complexity (non-granulocytes/granulocytes), and lack of expression of lineage markers (CD3^-^, CD19^-^, CD20^-^). **(B)** Difference in expression of CD16^+^ NK cells in liver compared to blood for a representative patient.

**Figure 2:**
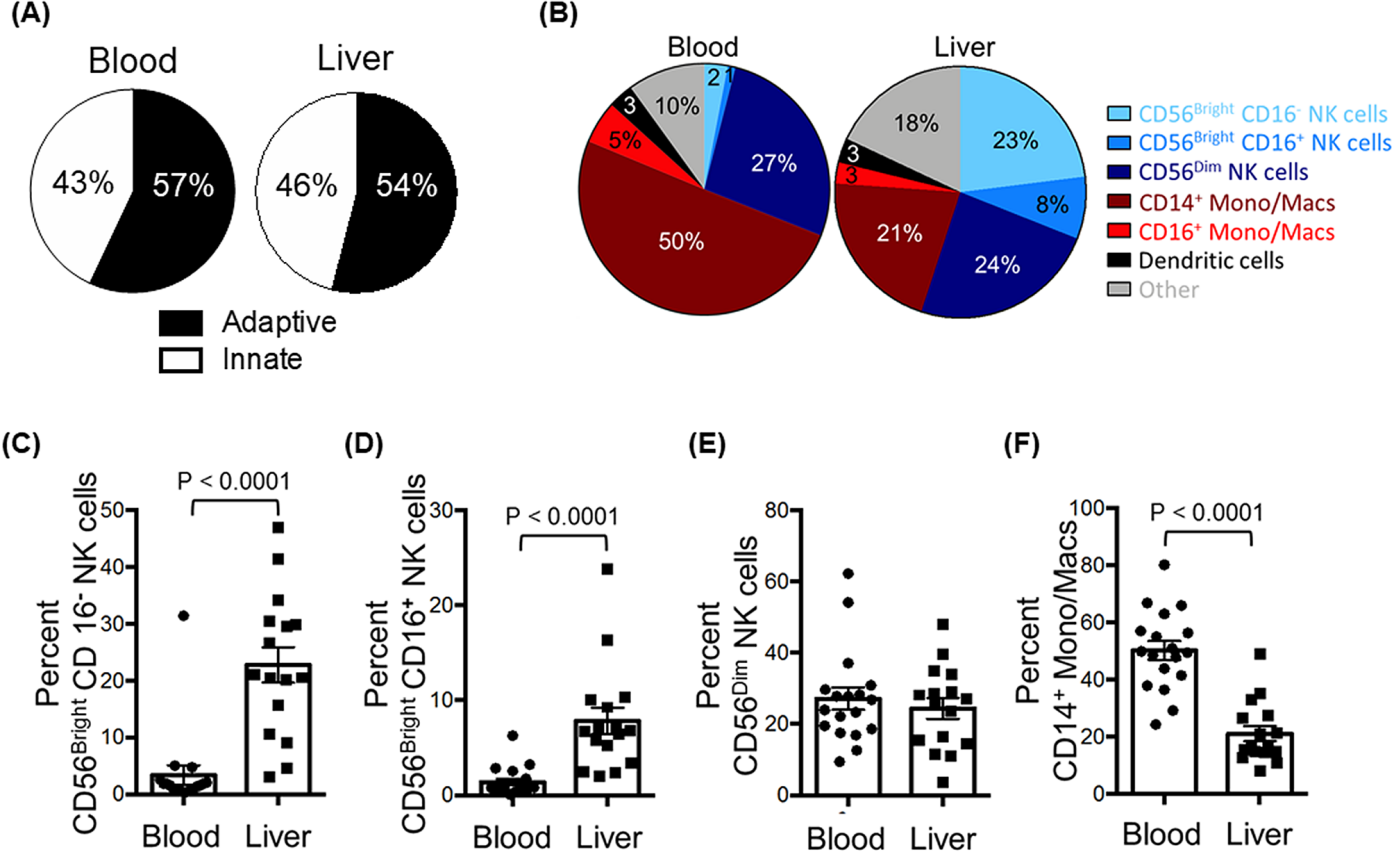
Comparison of the abundance of innate immune cells in the liver and in peripheral blood. **(A)** Pie charts depicting the composition of innate and adaptive immune cells from peripheral blood (left) and liver (right). **(B)** Pie charts depicting the composition of CD45^+^ lineage negative (CD3^-^, CD19^-^, CD20^-^) innate immune populations of the peripheral blood (left) and liver (right). **(C)** The percentage of CD56^Bright^ CD16^-^ NK cells of total hematopoietic (CD45^+^), lineage negative (CD3^-^, CD19^-^, CD20^-^) cells from liver and peripheral blood mononuclear cells. **(D)** The percentage of CD56^Bright^ CD16^+^ NK cells. **(E)** The percentage of CD56^Dim^ CD16^+^ NK cells. **(F)** The percentage of CD14^+^ monocytes/macrophages. Horizontal bars depict the mean ± SEM. N = 16 liver leukocytes, n = 18 PBMCs by unpaired *t* test.

We began our search for an immunoprotective cell subset by surveying liver and peripheral blood mononuclear cells from 19 HCV-infected patients undergoing liver transplantation, using the flow cytometry gating strategy in Fig. 1. The median age of the patients was 62 years [interquartile range (IQR), 59–65)]; 80% were male (Table 1). Innate immune cells (CD45^+^ CD3^-^ CD19^-^ CD20^-^) were a similar percentage of the CD45^+^ mononuclear cells in liver and peripheral blood: 46% in LMCs and 43% in PBMCs (Fig. 2A). Our multiparameter flow panel delineated three subsets of natural killer (NK) cells, two subsets of monocytes/macrophages, and three subsets of dendritic cells. The distributions of innate immune cells in liver and blood are presented in Fig. 2B–F.

**Table 1:** Characteristics of the study population.

Clinical characteristics, median (IQR), N (%)	Patients, N = 19
Age (years)	62 (59–65)
Male	15 (79%)
Hispanic	4 (21%)
Race	
Asian Black Caucasian Mixed Other	1 (5%) 3 (16%) 13 (68%) 1 (5%) 1 (5%)
Reason for liver transplantation	
Hepatocellular carcinoma End-stage liver disease	13 (68%) 6 (32%)
CMV IgG Ab	
Negative Positive	5 (26%) 14 (74%)
Natural MELD	18 (13–32) Range: 7–42
Total bilirubin (mg/dL) Normal: 0.1–1.2	2.9 (1.55–6.85) Range: 0.5–31
GGT (U/L) Normal: 9–48	48 (30–83) Range: 22–239
INR Normal: ≤ 1.1	1.55 (1.3–2.5) Range: 1.1–4.2
Creatinine (mg/dL) Normal: 0.6–1.2	1.13 (0.96–2.47) Range: 0.69–9.7
Albumin (g/dL) Normal: 3.4–5.4	3.3 (2.8–3.9) Range: 2.4–4.6
Platelets (× 10^3^/μL) Normal: 150–450	75 (57–95) Range: 21–146
White blood cell count (× 10^3^/μL) Normal: 4.5–11	4.65 (3.43–7.18) Range: 2.5–9.4

*IQR* interquartile range, *INR* international normalized ratio, *MELD* model for end-stage liver disease, *GGT* gamma-glutamyl transpeptidase.

NK cells were a higher percentage of the CD45^+^ innate immune mononuclear cells in liver than in blood. CD56^Bright^/CD16^-^ NK cells were an average of 23% in liver, but only 2% in blood, a highly-significant tenfold difference (*p* < 0.0001; Fig. 2B,C) that is consistent with published data^14^. The liver contained two additional NK subpopulations: CD56^Bright^/CD16^+^ NK cells, which were almost exclusively localized to liver (Fig. 2D; *p* < 0.0001) and CD56^Dim^/CD16^+^ NK cells, which had a similar abundance in liver and blood (Fig. 2E). CD14^+^ monocytes/macrophages were 50% of the innate immune mononuclear cells in blood, but only 21% in liver (Fig. 2F; *p* < 0.0001). CD16^+^ monocytes/macrophages were also enriched about two-fold in the blood (Fig. 2B; *p* = 0.02). Dendritic cells were a similar percentage of liver and blood innate immune cells; the compartmentalization of individual dendritic cell subsets (plasmacytoid, classical, and cross-presenting) was reported before^4^.

After comparing the two compartments based on the distribution of cell subsets, we compared them at the transcriptomic level. In this investigation of microarray data, we used gene set enrichment analysis (GSEA) and the blood transcriptome modules (BTM)^15^ to compare gene expression of freshly-isolated ex vivo cells from the liver and the blood. Three of the top five gene sets expressed at higher levels in LMCs were related to NK cells; the other two were related to T cells (Supplementary Table 1). Because three of the top five gene sets expressed at a higher level in LMCs were related to NK cells, we conclude that NK cells are more important in the hepatic microenvironment than in blood. Upregulated genes included multiple killer cell immunoglobulin-like receptor (KIR) genes^16^, and *EOMES* and *MYBL1*^17^, two transcription factors expressed in immature NK cells^18^. Additional upregulated genes encode granzymes A and K^19^, two proteases that are critical for NK function.

**Figure 3:**
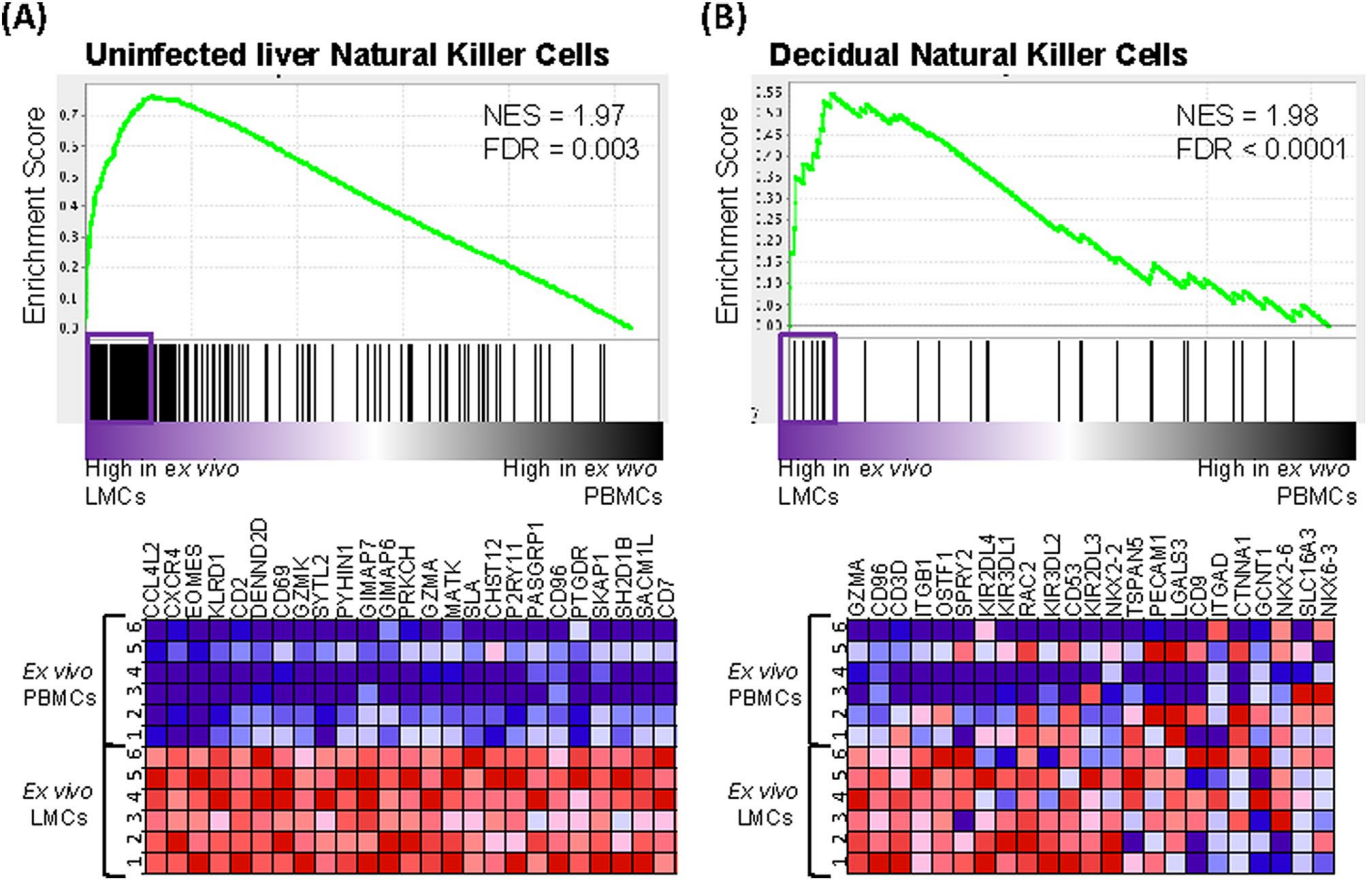
Liver mononuclear cells from HCV^+^ individuals are more similar to natural killer cells from healthy decidua and healthy liver. **(A)** GSEA of gene sets from Aizarani et al*.*^5^ related to “Uninfected Liver Natural Killer Cells” comparing ex vivo LMCs to ex vivo PBMCs from HCV-infected patients. **(B)** GSEA of gene sets from Koopman et al*.*^28^ related to “Decidual Natural Killer Cells” comparing ex vivo LMCs to ex vivo PBMCs. Pathways with a false discovery rate (FDR) below 0.25 were considered significant. Genes contributing to pathway enrichment (leading edge genes) are boxed and presented in the heatmaps.

The signatures of freshly isolated (ex vivo) LMCs and PBMCs were also compared to the single-cell RNA sequencing (scRNAseq) signature of liver NK cells from healthy liver organ donors analyzed by Aizarani et al*.*^5^ (Fig. 3A). The transcriptome of LMCs from the HCV-infected livers were significantly more similar to the signature of liver NK cells of organ donors (who do not have any underlying liver disease) than was the transcriptome of PBMCs from the HCV-infected patients (FDR = 0.003, Fig. 3A). The leading edge genes shared between LMCs of HCV-infected patients and uninfected liver NK cells included *EOMES* and *CD69*, a marker of tissue residency^14^. These data indicate that NK cells in the LMCs of HCV-infected patients retain key features of liver resident NK cells in uninfected patients; this important finding aligns with data of Cosgrove and colleagues^12^ who found, “the liver resident NK immunophenotype appeared unperturbed in the context of chronic HCV infection”.

### Hepatic immune cells have a blunted response to pro-inflammatory stimuli compared to blood

**Figure 4:**
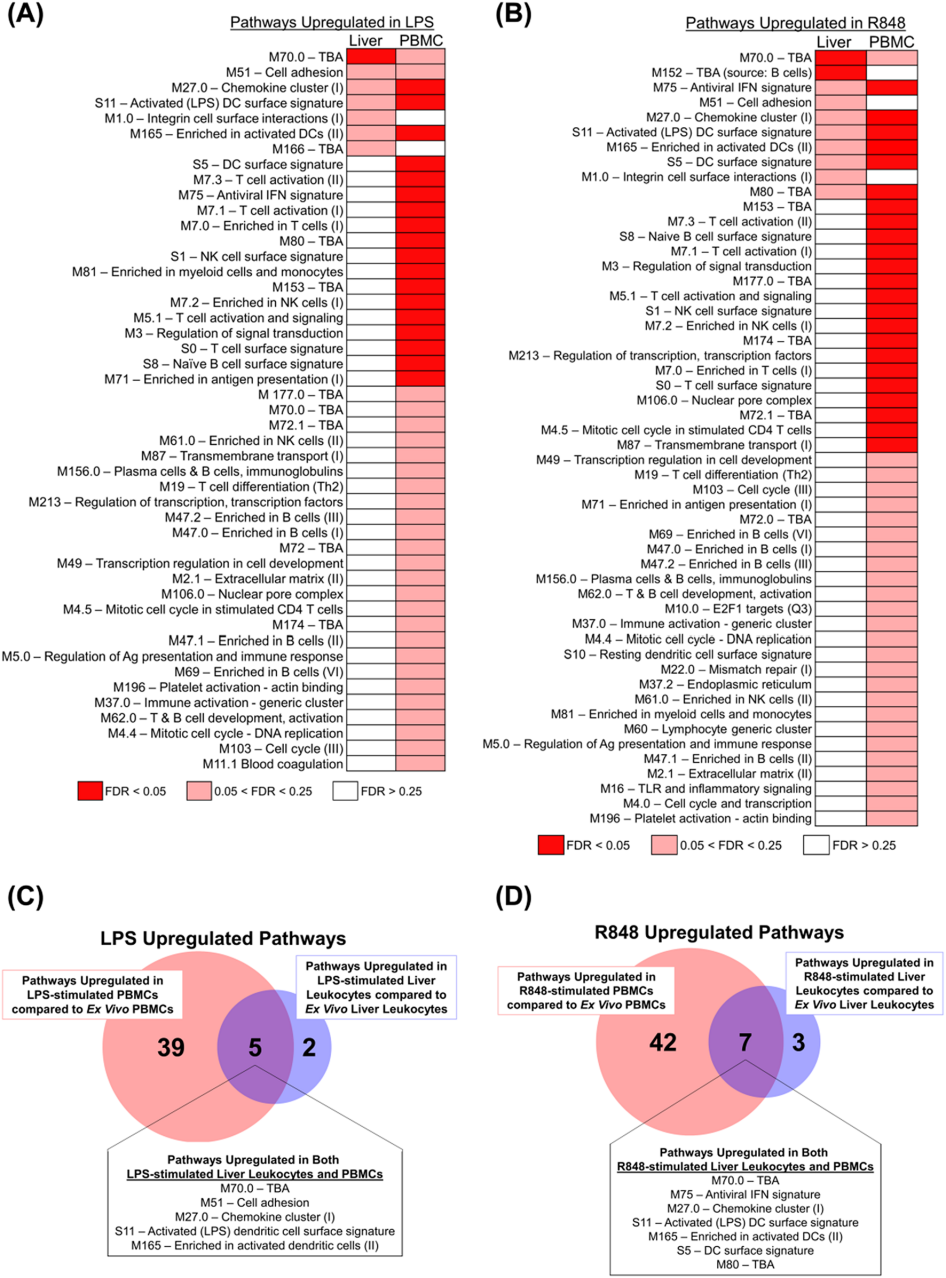
Fewer gene pathways are upregulated in stimulated LMCs than matched PBMCs. **(A)** Differential modulation of molecular pathways from the blood transcriptome modules (BTM) of ex vivo LMCs versus LPS-stimulated LMCs was compared to the modulation of molecular pathways from the same BTM pathways of ex vivo PBMCs versus LPS-stimulated PBMCs, analyzed by GSEA. **(B)** Differential modulation of molecular pathways from the BTMs of ex vivo LMCs versus R848-stimulated LMCs was compared to the modulation of molecular pathways from the same BTM pathways of ex vivo PBMCs versus R848-stimulated PBMCs, analyzed by GSEA. Pathways that were had a false discovery rate (FDR) of less than 0.05 are in red, between 0.05 and 0.25 FDR in pink, and greater than 0.25 FDR in white. **(C)** Shared BTM pathways upregulated with a FDR < 0.25 in both LPS-stimulated LMCs versus ex vivo LMCs and LPS-stimulated PBMCs versus ex vivo PBMCs. Leading edge genes driving these pathways are listed in Supplementary Table 2. **(D)** Shared BTM pathways upregulated with a FDR < 0.25 in both R848-stimulated LMCs versus ex vivo LMCs and R848-stimulated PBMCs versus ex vivo PBMCs. Leading edge genes driving these pathways are listed in Supplementary Table 3.

Pathogen associated molecular patterns (PAMPs) activate antimicrobial defenses and alter immune cell function. Compared to PBMCs, LMCs of HCV-infected patients are exposed to higher concentrations of HCV-related PAMPS and disease-associated molecular patterns (DAMPs) released from infected and damaged cells. Despite chronic exposure to these alarmins, the LMCs of the HCV-infected patients were far less responsive to LPS or the toll-like receptor (TLR)-7/8 agonist, R848, than PBMCs, as shown by the analysis of up-regulated gene expression pathways in Fig. 4. The LMC’s blunted response to LPS and to TLR 7/8 stimulation demonstrates that the liver’s well-known tolerogenic properties persist during HCV infection.

In this set of experiments, the pathways up-regulated by incubation in LPS (or R848) were first determined for the cells from each compartment (using FDR < 0.25 as the threshhold) and then the two sets of upregulated pathways were compared to each other. LPS induced 44 pathways in PBMCs, but only seven in LMCs (Fig. 4A,C). Similar to LPS, R848 up-regulated 49 pathways in PBMCs and only 10 pathways in LMCs (Fig. 4B,D). The leading edge genes of the pathways up-regulated in both LMCs and PMBCs are listed in Supplementary Table 2 (LPS-stimulation) and Supplementary Table 3 (R848-stimulation).

*IL22* was among the differentially expressed genes in cells exposed to LPS. It was more strongly up-regulated in LMCs than in PBMCs, *p* = 0.0002, FDR = 0.025. IL-22 stimulates repair processes in other tissues^20^ and, thus, our findings could indicate that LMCs are programmed to activate tissue repair pathways in response to pro-inflammatory stimulation^20^. In the liver, IL-22′s primary role is in tissue repair rather than inflammation^21^. Mice expressing *IL22* under an albumin promoter do not develop liver inflammation but are resistant to ConA T cell-mediated hepatitis. After partial hepatectomy, liver regeneration in these animals is accelerated through enhanced hepatocyte survival and proliferation^22^. Using single cell mass cytometry, we attempted to confirm that liver NK cells produce IL-22, as has been shown for other NK populations, but the antibody signal was too weak to identify IL-22-positive cells.

**Figure 5:**
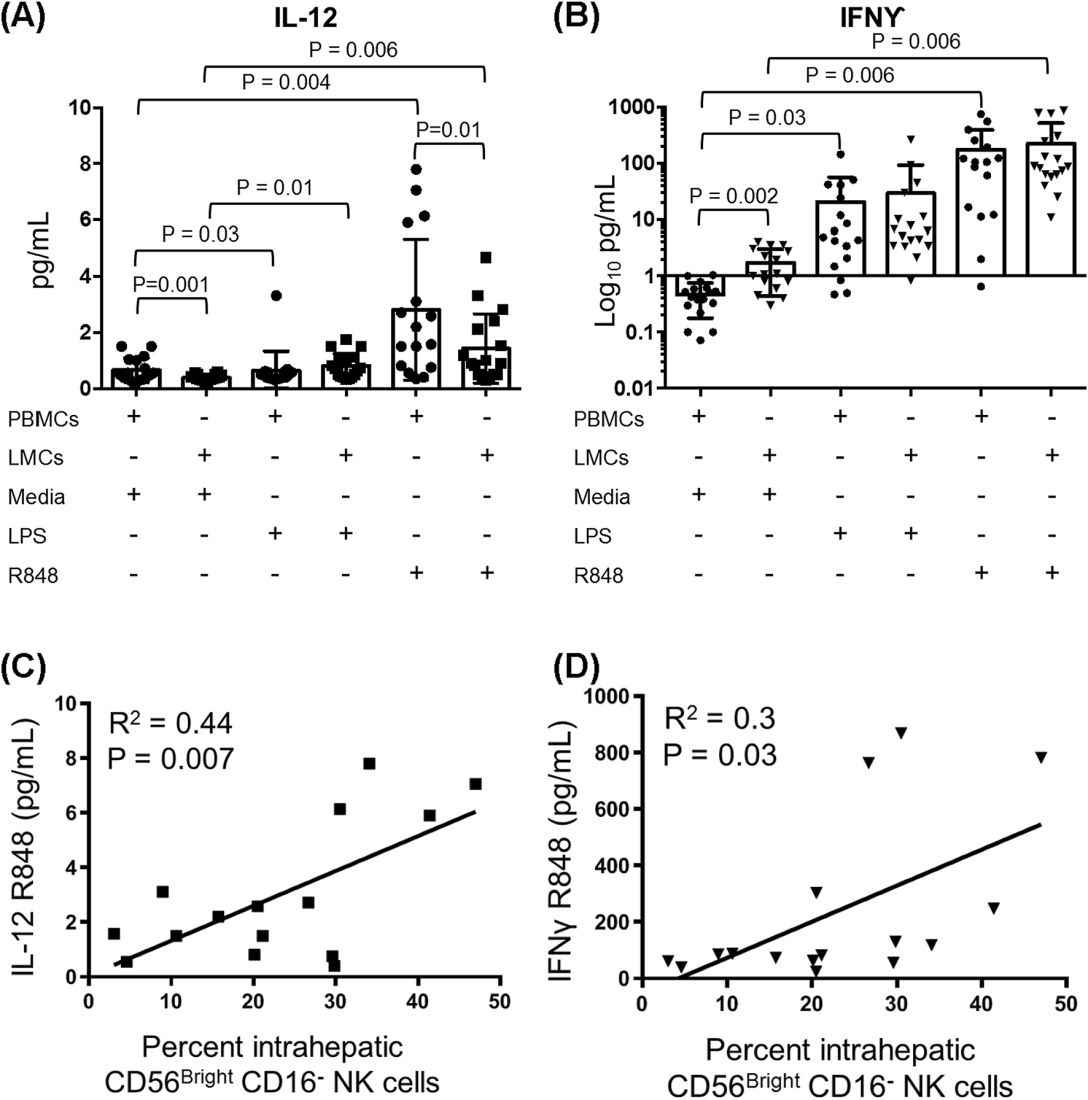
IFNγ secretion of TLR-stimulated LMCs is correlated with hepatic CD56^Bright^ CD16^-^ NK cells. LMCs and PBMCs were stimulated with LPS, R848 or media alone prior to analysis for cytokine production. Total pg/mL for **(A)** IL-12 and **(B)** IFNγ. Horizontal bars depict the mean ± SD. N = 16. Comparisons were made using paired t-tests. **(C)** IL-12 secreted from LMCs stimulated with R848 correlated with the percent hepatic CD56^Bright^ CD16^-^ NK cells. **(D)** IFNγ secreted from LMCs stimulated with R848 correlated with the percent hepatic CD56^Bright^ CD16^-^ NK cells. N = 16, calculated using the Pearson’s correlation coefficient.

To gain further insights into the effects of alarmins and to relate them to NK abundance in individual liver specimens, LMCs and PBMCs were incubated for 4 h in media, LPS or R848 and concentrations of the monokine, IL-12, and the cytokine, IFNγ, which are important for NK terminal differentiation and effector function, were measured in cell culture supernatants. The amount of IL-12 secreted by LMCs following R848 stimulation correlated with the prevalence of intrahepatic CD56^Bright^/CD16^-^ NK cells, as expected based on the well-known relationship between IL-12 and NK cells (Fig. 5A,C, *p* = 0.007). IFNγ produced by LMCs in response to TLR7/8 stimulation correlated with the abundance of CD56^Bright^/CD16^-^ NK cells (Fig. 5B,D, *p* = 0.03), suggesting that these cells contribute significantly to intrahepatic IFNγ production. LMCs incubated in media (with no TLR stimulation) secreted more of this anti-fibrotic cytokine than PBMCs (Fig. 5B), which may reflect the higher concentration of HCV RNA in liver tissue. These results suggest that liver resident NK cells may restrain liver fibrogenesis through the constitutive expression of IFNγ^23,24^.

### CD56^Bright^ CD16^-^ NK cells are uniquely correlated with indicators of better liver function

**Figure 6:**
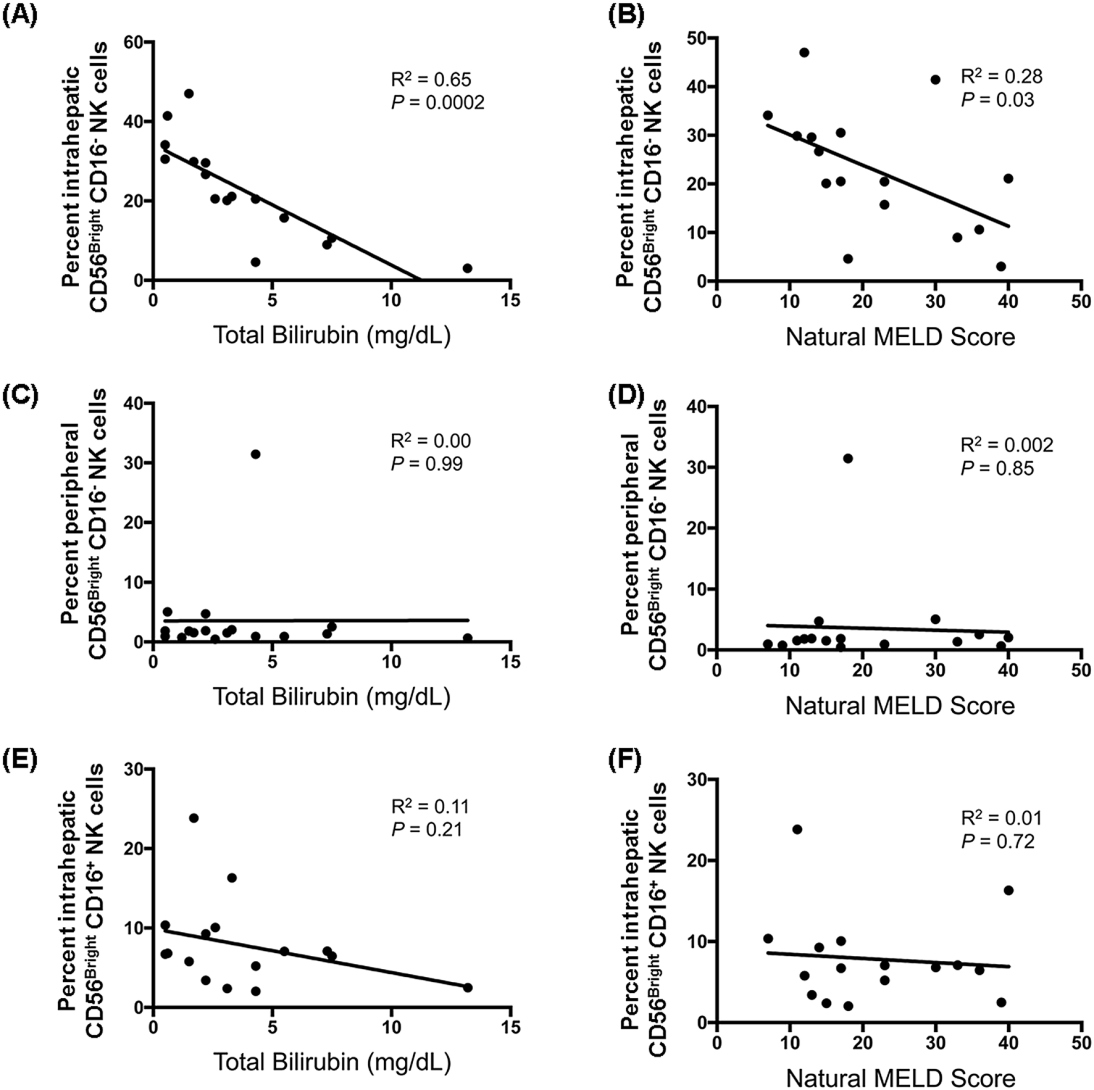
Correlation between the abundance of liver resident CD56^Bright^ CD16^-^ NK and better clinical status. The correlation between the abundance of hepatic CD56^Bright^ CD16^-^ NK cells and serum total bilirubin **(A)** and the natural MELD score calculated for the day of transplantation **(B)**. The correlation between the abundance of peripheral blood CD56^Bright^ CD16^-^ NK cells and serum total bilirubin **(C)** and the natural MELD score **(D)**. The correlation between the abundance of hepatic CD56^Bright^ CD16^+^ NK cells and serum total bilirubin **(E)** and the natural MELD score **(F)**. N = 16, calculated using the Pearson’s correlation coefficient.

We next examined the relationship between the prevalence of each of the sixteen innate immune cell populations (eight in LMCs and eight in PBMCs) and seven indicators of chronic liver disease severity. This analysis was possible because our study group included patients with a spectrum of liver disease severity: MELD scores ranged from 7 to 42, total bilirubin concentrations ranged from 0.5 to 31 mg/dL, and platelets ranged from 21 to 146 × 10^3^/μL (Table 1). The abundance of only one subset—hepatic CD56^Bright^/CD16^-^ NK cells—correlated with better liver function, as indicated by lower total serum bilirubin values (R^2^ = 0.65, *p* = 0.0002, Fig. 6A) and lower MELD scores (R^2^ = 0.28, *p* = 0.03, Fig. 6B). This association was highly specific for hepatic CD56^Bright^/CD16^-^ NK cells. No other subset of NK cells and none of the subsets of other innate immune cells had a statistically significant relationship with lower bilirubin and/or lower MELD scores. Bilirubin concentration was not correlated with blood CD56^Bright^/CD16^-^ NK cells (Fig. 6C, *p* = 0.99) or hepatic CD56^Bright^/CD16^+^ NK cells (Fig. 6E, *p* = 0.21), and MELD scores were not correlated with blood CD56^Bright^/CD16^-^ NK cells (Fig. 6D, *p* = 0.85) or hepatic CD56^Bright^/CD16^+^ NK cells (Fig. 6F, *p* = 0.72).

In contrast to the hepatic CD56^Bright^/CD16^-^ NK cells, greater abundance of hepatic CD16^+^/CD14^-^ monocytes/macrophages correlated with higher INR values (R^2^ = 0.42, *p* = 0.007, Supplementary Fig. 1A) and higher MELD scores (R^2^ = 0.35, *p* = 0.016, Supplementary Fig. 1B). Opposite, greater abundance of hepatic CD14^+^ monocytes/macrophages correlated with higher total bilirubin values (R^2^ = 0.27, *p* = 0.04, Supplementary Fig. 1C), consistent with published data showing that liver monocytes/macrophages promote liver inflammation^25^. Overall, hepatic CD56 ^Bright^/CD16^-^ NK cells emerged as the subset most likely to have a protective effect.

Single sample gene set enrichment analysis (ssGSEA) was used to investigate the impact of CD56^Bright^/CD16^-^ NK cells on the gene expression signature of surrounding tissue, with the expectation that the signature will be dominated by hepatocytes and the other major types of liver cells (endothelial cells, stellate cells, biliary epithelial cells. The ssGSEA enrichment score denotes the degree to which the genes in a particular pathway are upregulated in an individual specimen^26^. As was done before^13^, non-tumor tissue was used if a patients had hepatocellular carcinoma. ssGSEA showed that the abundance of CD56^Bright^/CD16^-^ NK cells correlated positively with two pathways associated with normal liver function (Supplementary Table 4): “Metabolism of porphyrins” (heme biosynthesis and breakdown are important hepatocyte functions) and “Circadian rhythm” (normal liver functions follow a pronounced circadian rhythm). Pathways with negative correlations included “Platelet adhesion to exposed collagen”, “Signaling by PDGF”, “Apoptotic executive phase” and “Apoptotic cleavage of cellular proteins”. Collectively, the ssGSEA analysis suggests that CD56^Bright^/CD16^-^ NK cells may promote liver homeostatic functions in surrounding cells. Consistent with the negative correlation with apoptotic pathways, a separate analysis showed that the abundance of intrahepatic CD56^Bright^/CD16^-^ NK cells did not significantly correlate with serum levels of HCV RNA (R^2^ = 0.19, *p* = 0.09), as might be expected if their beneficial effects are mediated by pathways that do not involve killing HCV-infected hepatocytes..

### Killer functions are inhibited in liver resident CD56^Bright^ CD16^-^ NK cells

The positive association between hepatic CD56^Bright^/CD16^-^ NK cells and liver homeostatic pathways suggested that they may have functional similarities to decidual NK cells, a key population that produces angiogenic factors and promotes vascular remodeling during the early phases of placental formation^27^. To test this, we compared a signature of decidual NK cells from Koopman et al*.*^28^ to the gene expression profiles of ex vivo LMCs and PBMCs. The LMCs were much more similar to the decidual NK cells than were the PBMCs (FDR < 0.0001, Fig. 3B). Several of the leading edge genes encode proteins that *inhibit* NK functions, including *KIR2DL4*, *KIR3DL1*, *CD53*, *KIR2DL3*^16^, and *SPRY2*^29^, suggesting that liver resident CD56^Bright^/CD16^-^ NK cells are primed to resist activation of killing pathways.

**Figure 7:**
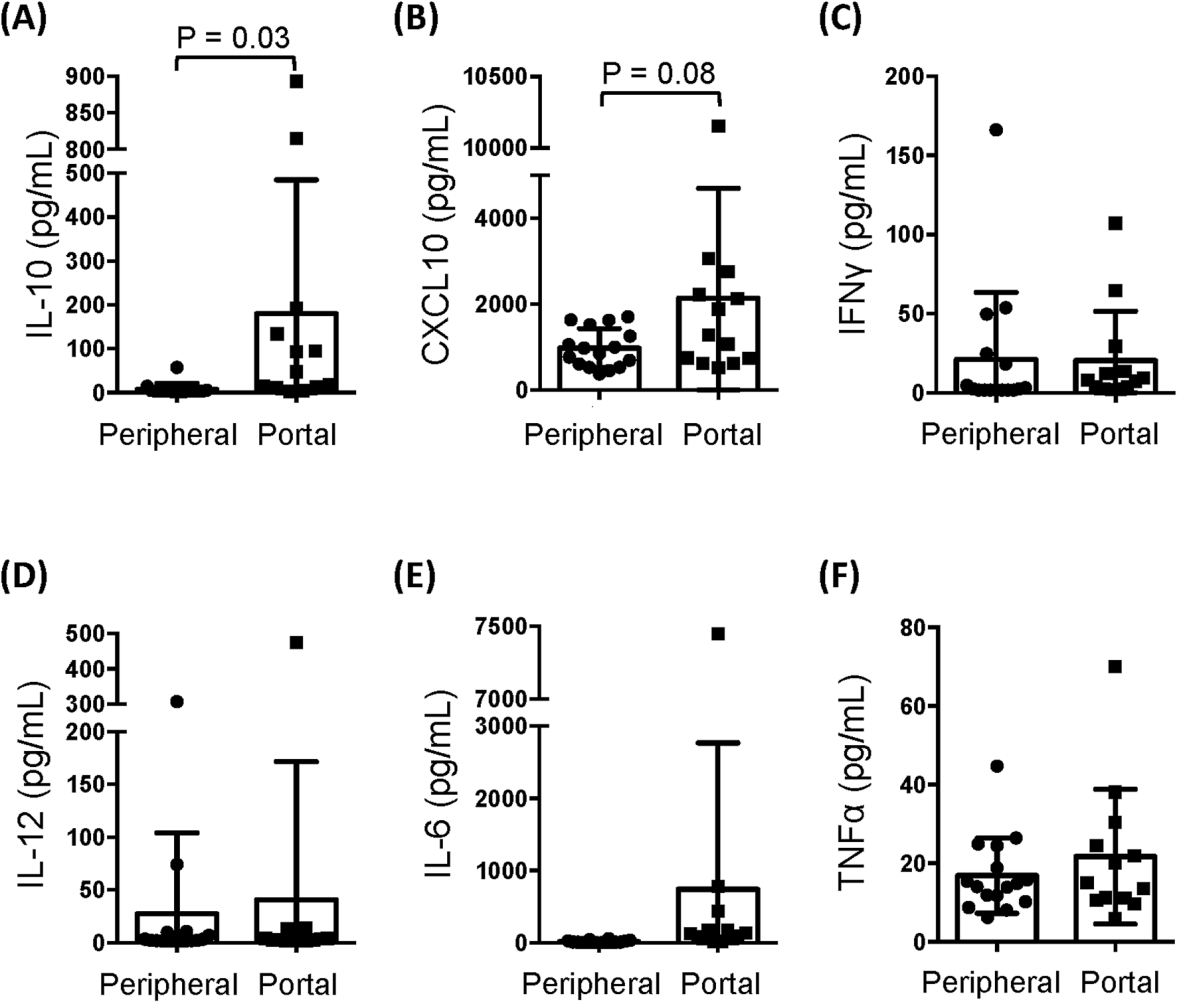
Portal vein blood has a higher concentration of IL-10 than peripheral blood. Portal vein plasma and peripheral plasma was analyzed for concentrations of **(A)** IL-10, **(B)** CXCL10, **(C)** IFNγ, **(D)** IL-12, **(E)** IL-6, and **(F)** TNFα. Horizontal bars depict the mean ± SD. N = 16 peripheral plasma, n = 13 portal vein plasma by unpaired *t* test.

Because the portal vein supplies the majority of blood to the liver, cytokines in portal vein blood may influence the programming of the hepatic immune response. Importantly, the concentration of IL-10, an immunosuppressive factor, was significantly higher in portal vein plasma than in peripheral blood plasma (Fig. 7A), while concentrations of the other cytokines (CXCL10, IFNα, IFNγ, IL-6, IL-12, TNFα) were similar in the two compartments (Fig. 7B–F). The high concentration of IL-10 in portal vein blood could prime LMCs for a blunted and tolerogenic response to pro-inflammatory TLR ligands.

## Discussion

This study revealed a strong association between the abundance of human liver CD56^Bright^/CD16^-^ NK cells and measures liver function, such as lower total bilirubin concentration in serum, lower MELD scores, and expression of molecular pathways associated with normal liver functions. The abundance of liver CD56^Bright^/CD16^-^ NK cells accounted for 65% of the variance in total bilirubin in serum. In contrast to the NK cells, the abundance of hepatic monocytes/macrophages was associated with increased liver injury and dysfunction, consistent with published data implicating these cells in liver damage. Monocyte recruitment and expansions in liver accelerate fibrogenesis in rat and mouse models^30^; and the number of CD14- and CD16-expressing hepatic cells is positively correlated with scar tissue and liver damage^25^. While hepatic monocytes/macrophages cause pro-inflammatory liver damage, hepatic CD56^Bright^/CD16^-^ NK emerged from our data as the subset of innate immune cells most likely to have a protective effect. Cosgrove and colleagues reached a similar conclusion. They also found a strong inverse relationship between serum bilirubin and intrahepatic CD56^Bright^ NKG2D^+^ NK cells (r = − 0.861) and proposed that NK cells attenuate liver fibrogenic pathways in chronic HCV infection^12^.

Our findings cconfirm previous data showing that CD56^Bright^ NK cells are highly enriched in liver compared to blood^12,14,31–35^ and they suggest several mechanisms hepatic CD56^Bright^/CD16^-^ NK cells might use to promote liver homeostasis and repair. The main cytokines NK cells are known to produce are IFNγ, TNFα, and IL-10^36^. Of these, our data support a role for IFNγ. Without TLR stimulation, liver leukocytes produced more IFNγ than PBMCs, suggesting that the liver cells are primed for spontaneous secretion. The amount of IFNγ produced by LMCs in response to TLR7/8 stimulation correlated with the abundance of hepatic CD56^Bright^/CD16^-^ NK cells, indicating that these cells may be a major source of hepatic IFNγ. In other settings, IFNγ has antifibrotic effects and is part of regulatory circuits that down-modulate pathways of tissue injury^37^. IFNγ from liver NK cells may contribute to analogous processes in liver.

In addition to a possible antifibrotic effect of IFNγ, the positive association between hepatic CD56^Bright^/CD16^-^ NK cells and liver homeostatic gene expression pathways suggested that these cells may have functional similarities to decidual NK cells, a key population that produces angiogenic factors and promotes vascular remodeling during the early phases of placental formation^27^. The signature of decidual NK cells from Koopman et al.^28^ had several genes in common with that of liver NK cells from our patients. A number of the shared leading edge genes encode proteins that *inhibit* NK functions, including *KIR2DL4*, *KIR3DL1*, *CD53*, *KIR2DL3*, and *SPRY2*, indicating that the liver resident CD56^Bright^/CD16^-^ NK cells may be primed to resist activation of killing pathways. CD56^Bright^/CD16^-^ NK cells express low levels of perforin and they have low cytotoxicity^36^. Taken together with published data showing that liver NK abundance is inversely related to serum markers of hepatocyte damage, ALT and AST^11^, our results indicate that hepatic CD56^Bright^/CD16^-^ NK cells preserve liver function through mechanisms independent of cell killing. Interestingly, decidual NK cells produce IFNγ constituitively, suggesting that this cytokine may contribute to an essential function of the resident NK cells in these two compartments.

After LPS stimulation, LMCs up-regulated expression of *IL22*. Because IL-22 is associated with liver regeneration in other settings^20^, this up-regualtion could indicate that the LMCs are primed to up-regulate tissue repair pathways in response to pro-inflammatory stimulation^20^. Further investigation is needed to determine whether liver NK cells secrete this cytokine. Additional mechanisms liver NK cells might use to promote liver function could include an enhancement of hepatocyte survival and proliferation^21^, stimulation of liver repair^38^, down-modulation of pro-inflammatory responses, and killing hepatic stellate cells^39^.

Portal vein blood could play a role in the programming of liver NK cells for tissue repair. The liver receives most of its blood directly from the gut vasculature through the portal vein, and thus hepatic cells are exposed to high levels of food antigens and bacterial PAMPS, including LPS. The liver must clear these non-self antigens without becoming inflamed, while also maintaining antimicrobial defenses. The liver immune system helps achieve the balance necessary for homeostasis. The tolerogenic nature of the liver innate immune system is due to lower levels of TLRs^40^, cytokine-mediate suppression^41^, liver microenvironment reprograming^42^, and enrichment of tolerogenic innate immune populations^43^. In this study of HCV-infected patients, we found that liver leukocytes were far less responsive to TLR stimuli than PBMCs, demonstrating that the liver’s hypo-responsiveness to pro-inflammatory stimuli continues during HCV infection. The high concentration of IL-10 in portal vein blood may help establish and maintain this hypo-responsiveness. IL-10 is important for maintaining NKG2A^+^ NK cells in a hypo-responsive state that promotes the induction of T regulatory cells^44^. It is thus plausible that IL-10 in portal vein blood modulates liver NK cells and down-regulates their cytototoxic activities.

The study has several strengths. It is a prospective investigation using specimens that were processed in an expedited manner to preserve cell viability. The sample size of 19 is robust for a study of this depth. Multiparameter flow cytometry allowed eight subsets of hepatic innate immune cells to be delineated and compared to PBMCs. Finally, the flow cytometry data were integrated with clinical, molecular, and secretomic analyses. The study also has limitations. All the patients had HCV infection; however, Cosgrove and colleagues compared NK cells from patients with and without HCV infection and found that “the profile of intrahepatic NK cells was remarkably unaltered in chronic HCV infection” ^12^, and we found an association between the gene expression profile of LMCs and liver NK cells of patients with no underlying liver disease^5^. Additionally, our experiments did not prove that there is a causal relationship between the abundance of liver NK cells and better liver function. Our results allow other interpretations: CD56^Bright^/CD16^-^ NK cells could be a non-functional precursor population, for example. Despite these limitations, our study achieved its primary objective: Delineating eight innate immune cell subsets in human liver and blood and identifying the one that is most strongly associated with maintenance of normal liver homeostatic functions.

In summary, our findings suggest that hepatic CD56^Bright^/CD16^-^ NK cells may be a source of immunoprotective factors in the liver. These cells may counter the inflammatory effects of macrophages, which would otherwise cause excessive liver damage, and they may share functions with decidual NK cells. The hyporesponsiveness of liver innate immune cells to TLR4 or 7/8 stimulation is maintained in patients with a chronic viral infection, despite inflammation and activation of IFN-stimulated antiviral defense pathways^12,45^.

## Methods

### Study design

This is a prospective study of 19 HCV-positive adults who underwent liver transplantation at the Mount Sinai Medical Center between November 2013 and August 2014. Informed consent was obtained in writing from patients before transplantation. The study protocol was approved by the Icahn School of Medicine at Mount Sinai’s institutional review board and adhered to all its guidelines. Medical records were reviewed for clinical/demographic data.

### Specimen collection

Blood for research and for clinical testing was collected prior to surgery and liver explant tissue and portal vein blood were obtained at the time of surgery. After the recipient hepatic veins and arteries were clamped, portal vein blood was collected and used to prepare portal vein plasma. Liver explants were accessed by liver pathologists in an expedited manner to allow cell preparation to begin as quickly as possible, optimizing cell viability. Liver tissue arrived at the cell isolation laboratory within a median of 45 min of surgery. Tissue was processed, as described before^4^. Mononuclear cells were isolated using a percoll gradient.

### Flow cytometry

Nine-color multiparameter flow cytometry was used to delineate cell subsets using the antibodies reported previously^4^ for CD45, CD3, CD19, CD20, HLA-DR, CD14, CD16, CD123, BDCA1, BDCA3, and CD56, using the gating strategy in Fig. 1. NK cells were defined as CD45^+^ CD3^-^ CD19^-^ CD20^-^ HLA-DR^-^ CD14^-^ and classified in 3 subsets based on CD56 and CD16 expression: CD56^Bright^/CD16^-^, CD56^Bright^/CD16^+^, and CD56^Dim^/CD16^+^. Subpopulations of monocytes/macrophages were defined by the following markers: CD45^+^ CD3^-^ CD19^-^ CD20^-^ HLA-DR^+^ CD14^+/-^ CD16^+/-^.

### Cell stimulation in vitro, proteomics, and RNA extraction

Liver mononuclear cells (LMCs) and peripheral blood mononuclear cells (PBMCs) were stimulated with two toll-like receptor (TLR) agonists [200 ng/mL lipopolysaccharide (LPS) or 1 μg/mL resiquimod (R848), a TLR7/8 agonist] or incubated in media for four hours at 37 °C. Supernatants were collected for secretomic analysis. Luminex multiplex cytokine assays (Millipore) quantified interferon (IFN) gamma-induced protein 10 (IP10 aka CXCL10), interleukin 6 (IL-6), IL-10, IL-12p70, IFNγ and tumor necrosis factor (TNF)α. Cells were collected in Trizol (Life Technologies) and RNA was purified using RNeasy mini kits (Qiagen), according to manufacturer’s instructions^4^.

### Microarray analysis

Profiling data from Illumina Human-HT-Expression Beadchips were normalized using GenomeStudio’s quantile method. GenePattern was used for gene set enrichment analysis (GSEA) and single sample GSEA (ssGSEA) of immune pathways^46^ using blood transcriptomic modules (BTMs)^15^, KEGG pathways^47^, and Reactome pathways^48^. A false discovery rate (FDR) below 0.25 was considered statistically significant based on the Broad Institute’s guidelines^49^ Comparative Marker Selection (GenePattern) was used to determine the genes that were most differentially expressed. LMCs/PBMCs: To obtain sufficient RNA, LMC samples of matched pairs of patients were pooled. Matching was based on age, gender, HCV genotype, baseline HCV RNA, natural MELD score, and HCC (yes/no). PBMCs were pooled similarly. Whole liver: Whole liver microarray data of 11 of the 19 patients consented for this analysis was used, as before^4^.

### Statistical analysis

GraphPad Prism was used for statistical analysis. T-tests, both paired and unpaired, were performed. Linear regression was used for clinical correlations.

## Supplementary Information

Supplementary Tables.

Supplementary Figure S1.

## Abbreviations

ALT: Alanine aminotransferase
CD: Cluster differentiation
GGT: Gamma-glutamyl transferase
HBV: Hepatitis B virus
HCV: Hepatitis C virus
IFN: Interferon
IL: Interleukin
INR: International normalized ratio
IQR: Interquartile range
LMC: Liver mononuclear cell
LPS: Lipopolysaccharide
MELD: Model of end-stage liver disease
NK: Natural killer
PAMPS: Pathogen associated molecular patterns
PBMC: Peripheral blood mononuclear cell
TLR: Toll-like receptor
TNFα: Tumor necrosis factor alpha
