# Supplementary material for: A subset of liver resident natural killer cells is expanded in hepatitis C-infected patients with better liver function
Source: Sci Rep. 2021 Jan 15;11:1551. doi: 10.1038/s41598-020-80819-8 (PMC7810844; doi:10.1038/s41598-020-80819-8)
Supplement: Supplementary file 1 — Supplementary Tables. [file 41598_2020_80819_MOESM1_ESM.docx]

**Title:** A Subset of Liver Resident Natural Killer Cells Is Expanded in Hepatitis C-infected Patients with Better Liver Function

**Authors**:

Erin H. Doyle^1^, Costica Aloman^2^, Ahmed El-Shamy^1^, Francis Eng^1^, Adeeb Rahman^3^, Arielle L. Klepper^1^, Brandy Haydel^4^, Sander S. Florman^4^, M. Isabel Fiel^5^, Thomas Schiano^4^, and Andrea D. Branch^1^

^1^Division of Liver Diseases, Icahn School of Medicine at Mount Sinai School, New York, NY

^2^Rush University Medical Center, Chicago, IL

^3^Human Immune Monitoring Core, Icahn School of Medicine at Mount Sinai, New York, NY

^4^Recanati Miller Transplantation Institute, The Mount Sinai Hospital, New York, NY

^5^Department of Pathology, The Mount Sinai Hospital, New York, NY

**Supplemental Figure 1. Correlation between macrophage/monocyte populations and clinical status.** Correlation between the abundance of hepatic CD16^+^ CD14^-^ monocytes/macrophages were correlated with INR (A) and the natural MELD score (B). Correlation between the abundance of hepatic CD14^+^ monocytes/macrophages and serum total bilirubin (C). N=16, calculated using the Pearson’s correlation coefficient.

| **Suppletory Table 1. Top 5 gene sets enhanced in LMCs compared to PBMCs** | | | |
| --- | --- | --- | --- |
| **Gene set** | **NES** | **FDR** | **Top genes of leading edge subset** |
| S1 – NK cell surface signature | 1.96 | 0.006 | *TNFSF14, TGFBR3, PTGDR, IL2RB, ADRB2, FASLG, IL18RAP, HAVCR2, IL18R1, S1PR5, RARRES3, ATP8B4, GPR114, CD97, KIR3DL1, KIR2DL3, KIR2DS5, KIR2DL1, CX3CR1, SLC7A5* |
| S0 – T cell surface signature | 1.93 | 0.006 | *CD2, PTPRCAP, CXCR6, CD6, GIMAP2, ITM2A, SLC38A1, LAG3, GPR171, CD3E, SIT1, TMEM106C, CLEC2D* |
| M7.2 – Enriched in NK cells (I) | 1.93 | 0.007 | *EOMES, KLRD1, TARP, GIMAP7, PRKCH, GZMA, CD96, PTGDR, IL2RB, MYBL1, CD247, FASLG, PLEKHF1, IL18RAP, KLRC1, FCGR3B, PRKCQ, CST7, GNLY, PRF1, ITK, CCL5, S1PR5, CLIC3, SH2D2A, GZMB, SAMD3, NLRC3, GPR56, HOPX, GZMH, FGFBP2, NKG7, CTSW* |
| M7.0 – Enriched in T cells (I) | 1.96 | 0.008 | *EOMES, TARP, CD2, GZMK, GIMAP7, GIMAP6, PRKCH, GZMA, RASGRP1, CD96, CD7, CD247, PTPRCAP, CD6, PRKCQ, CD3D, UBASH3A, GNLY, PRF1, ITK, CCL5, ITM2A, BCL11B, GZMB, SAMD3, GPR171, NLRC3, IL32, CD3E, SIT1, GZMH, NKG7, LAT, TRAT1* |
| M61.0 – Enriched in NK cells (II) | 1.94 | 0.008 | *TARP, TGFBR3, CD96, CD7, IL2RB, CD247, S1PR5, GPR56, KIR3DL1, KIR2DL3, KIR2DS5, KIR2DL1, NKG7* |

| **Supplementary Table 2. Leading edge genes for shared LPS upregulated pathways** | | | | | | |
| --- | --- | --- | --- | --- | --- | --- |
|  | **Upregulated in LPS-stimulated LMCs compared to *Ex Vivo* LMCs** | | | **Upregulated in LPS-stimulated PBMCs compared to *Ex Vivo* PBMCs** | | |
| **Gene set** | **NES** | **FDR** | **Top genes of leading edge subset** | **NES** | **FDR** | **Top genes of leading edge subset** |
| M70.0 – TBA | 1.99 | 0.006 | *MSC, IL4I1, CFB, PDLIM7, NUP62* | 1.61 | 0.066 | *CFB, IL4I1, NUP62, MSC, CES4, ZNF205* |
| M51 – Cell adhesion | 1.58 | 0.124 | *IL1B, ENG, VCAM1, THBS1, COL5A1, JAM3* | 1.48 | 0.121 | *THBS1, IL1B, ENG, LAMB1, JAM3* |
| M27.0 – Chemokine cluster (I) | 1.61 | 0.178 | *CCL7, CCL20, CCL23, CCL4, CXCL5, CXCL1, CXCL2, CCL19, CCL8, CXCL9, CXCL13, CCL18, CXCL3, PPBP* | 2.10 | 0.001 | *CCL20, CXCL2, CXCL5, CCL7, CXCL1, CCL8, CXCL13, CCL23, CCL4, CXCL6, CXCL9, CCL19* |
| S11 – Activated (LPS) DC surface signature | 1.61 | 0.132 | *RNF144B, CD40, TMEM140, ITGB8, EREG, SLC41A2, SGPP2, LRRC32, IL3RA, IFI27, CD58, SLC43A2* | 1.87 | 0.008 | *RNF144B, EREG, TMEM140, ITGB8, IFI6, CD58, SGPP2, CD40, TGFA, SLC43A2, SLC31A2, TNFSF13B, LAMP2* |
| M165 – Enriched in activated DCs (II) | 1.50 | 0.122 | *HERC5, IFIH1, TNFAIP6, CCL20, RSAD2, IFIT2, LAMP3, MX2, DSE, IFIT3, IFI27, CCL8, SERPING1, IL18, IFIT1, PDGFRL, HESX1, DAPK1, IFNGR2, MGST1, ST3GAL6* | 2.11 | 0.001 | *CCL20, TNFAIP6, HERC5, IFIH1, DSE, CCL8, IFIT2, MX2, RSAD2, RBM47, LAMP3, PDGFRL, IFNGR2, CSF2RA, IFIT3, IFIT1, DAPK1, IL18* |

| **Supplementary Table 3. Leading edge genes for shared R848 upregulated pathways** | | | | | | |
| --- | --- | --- | --- | --- | --- | --- |
|  | **Upregulated in R848-stimulated LMCs compared to *Ex Vivo* LMCs** | | | **Upregulated in R848-stimulated PBMCs compared to *Ex Vivo* PBMCs** | | |
| **Gene set** | **NES** | **FDR** | **Top genes of leading edge subset** | **NES** | **FDR** | **Top genes of leading edge subset** |
| M70.0 – TBA | 1.86 | 0.013 | *IL4I1, MSC, CFB, PDLIM7, NUP62* | 1.56 | 0.081 | *IL4I1, CFB, NUP62, MSC* |
| M75 – Antiviral IFN signature | 1.53 | 0.082 | *RSAD2, HERC5, OAS1, OAS3, DHX58, IL1B, IRF7, DDX58, DDX60, PTX3, BCL3, SERPING1, IFIT1* | 1.85 | 0.016 | *RSAD2, HERC5, DDX60, DDX58, DHX58, OAS1, IL1B, OAS3, IRF7, IFIT1, PTX3, BCL3, SERPING1* |
| M27.0 – Chemokine cluster (I) | 1.46 | 0.141 | *CCL8, CXCL9, CCL7, CCL4, CCL20, CXCL13, CCL19, CXCL2, CXCL1, CCL23, CXCL5, CCL18, PPBP* | 2.07 | 0.002 | *CCL8, CCL7, CCL20, CXCL2, CXCL1, CXCL9, CXCL5, CXCL13, CCL23, CCL19, CCL4, CCL1* |
| S11 – Activated (LPS) DC surface signature | 1.45 | 0.144 | *TMEM140, IFI27, SLC41A2, RNF144B, CD40, LRRC32, IFI6, ITGB8, SGPP2, IL3RA, SLC31A2, SLC43A2, CD58, EREG* | 2.01 | 0.003 | *TMEM140, RNF144B, CD40, ITGB8, IFI6, SLC31A2, TGFA, TNFSF13B, IFI27, LAMP2* |
| M165 – Enriched in activated DCs (II) | 1.53 | 0.082 | *CCL8, RSAD2, LAMP3, HERC5, IFIH1, MX2, TNFAIP6, IFIT2, IFI27, SERPING1, HESX1, IFIT3, CCL20, PDGFRL, IFIT1, DSE, HLX* | 2.06 | 0.002 | *CCL8, RSAD2, HERC5, IFIH1, IFIT2, TNFAIP6, CCL20, MX2, PDGFRL, LAMP3, DSE, HESX1, RBM47, IFIT1, IFIT3, SERPING1, HLX, IFNGR2, CSF2RA, IL18, IFI27* |
| S5 – DC surface signature | 1.42 | 0.170 | *LHFPL2, CD83, SLC41A2, OLR1, GRINA, TFRC, ITPRIPL2, TSPAN33, GJB2, JAG1, KMO, HLADQA1, SLC7A11, CXCL16, CLDN23, SLC6A6, TMEM158, CD58, SDC2, NRP1, TM2D2, C19orf28, PSEN2* | 2.13 | 0.003 | *OLR1, LHFPL2, SDC2, ITPRIPL2, TFRC, GJB2, CLDN23, SLC7A11, CD83, HLADQA1, CD58, GRINA, CXCL16, SLC1A3, NRP1, ATP1B1, TGFA, GPNMB, SLC6A6, LAMP2, PPAP2B, RAMP1, TMEM158, P2RY6, FPR3, FZD5* |
| M80 – TBA | 1.36 | 0.243 | *GPR132, RGL1, MARCH3* | 1.90 | 0.009 | *RGL1, GPR132, HCP5, MARCH3, PTK2, CTGF, TRAT1, ENG, FAM43A, GNG11* |

| **Supplementary Table 4. Intrahepatic CD56^Bright^ CD16^-^ NK cell correlations with whole liver gene pathways.** | | | | |
| --- | --- | --- | --- | --- |
| **Pathway** | **Slope**  **Direction** | **R^2^** | **P Value** | **Relationship to function** |
| **Positive Slopes** |  |  |  |  |
| Reactome – Metabolism of porphyrins | Positive | 0.64 | 0.003 | Heme biosynthesis and breakdown is a normal function of hepatocytes. |
| KEGG – Circadian_Rhythm_Mammal | Positive | 0.50 | 0.017 | Liver functions follow a circadian rhythm |
| **Negative Slopes** |  |  |  |  |
| Neuro Transmitters and Pathways |  |  |  |  |
| Reactome- Neurotransmitter  Receptor Binding and Signaling | Negative | 0.92 | <0.0001 | Altered neurotransmission may contribute to sleep disturbances |
| Reactome- GABA Receptor Activation | Negative | 0.70 | 0.0013 | γ-aminobutyric acid (GABA) is both a neurotransmitter and signals through receptors on liver cells |
| Reactome-Opioid Signaling | Negative | 0.69 | 0.005 | Increased opioid signaling is associated with itch in primary biliary cholangitis |
| PLC Signaling Pathway |  |  |  |  |
| Reactome – Ca^2+^ dependent events | Negative | 0.75 | 0.0006 | Increased Ca^2+^ in the hepatocyte cytosol reduces bile secretion^43^ |
| Reactome – DAG and IP3 signaling | Negative | 0.73 | 0.0008 | DAG and IP3 are downstream products of PLC and induce Ca^2+^ |
| Reactome – PLC beta mediated events | Negative | 0.71 | 0.0011 | PLC creates DAG and IP3 to influence Ca^2+^ |
| Reactome – Phospholipase C mediated cascade | Negative | 0.62 | 0.0038 | PLC creates DAG and IP3 to influence Ca^2+^ |
| Hyperglycemia |  |  |  |  |
| Reactome – Glucagon signaling in metabolic regulation | Negative | 0.67 | 0.002 | Increased glucagon signaling and hyperglycemia are caused by inflammation and poor health^44^ |
| Reactome – Synthesis of PA (phosphatidic acid) | Negative | 0.64 | 0.003 | Increased in phosphatidic acid production leads to hyperglycemia^45^ |
| Reactome – Regulation of insulin secretion by glucagon-like peptide 1 | Negative | 0.56 | 0.0084 | GLP 1 is induced by hyperglycemia in hopes to regulate glucagon |
| **Negative Liver Functions** |  |  |  |  |
| Reactome – Acyl chain remodeling of PG (phosphatidylglycerol) | Negative | 0.71 | 0.0011 | Phosphatidylglycerol is remodeled more in steatosis and cirrhosis^46^ |
| Reactome – Platelet adhesion to exposed collagen | Negative | 0.65 | 0.0027 | Type I collagen in upregulated in liver fibrosis^47^ |
| Reactome – Acyl chain remodeling of PI (phosphatidylinositol) | Negative | 0.61 | 0.0047 | Dietary phosphatidylinositol prevents NASH in mice^48^ |
| Reactome – Activated NOTCH1 transmits signal to the nucleus | Negative | 0.59 | 0.0056 | NOTCH1 signaling is important in HCC^49^ |
| Reactome – Signaling by PDGF | Negative | 0.52 | 0.0056 | PDGF signaling is enhanced in fibrosis^50^ and HCC^51^ |
| **Apoptosis** |  |  |  |  |
| Reactome – Apoptotic execution phase | Negative | 0.64 | 0.003 | Reduced liver apoptosis with more CD56^Bright^ CD16^-^ NK cells |
| Reactome – Apoptotic cleavage of cellular proteins | Negative | 0.61 | 0.0043 | Reduced interactions between cells and extracellular matrix proteins |
