# Supplementary figures and images for: A subset of liver resident natural killer cells is expanded in hepatitis C-infected patients with better liver function
Source: Sci Rep. 2021 Jan 15;11:1551. doi: 10.1038/s41598-020-80819-8 (PMC7810844; doi:10.1038/s41598-020-80819-8)

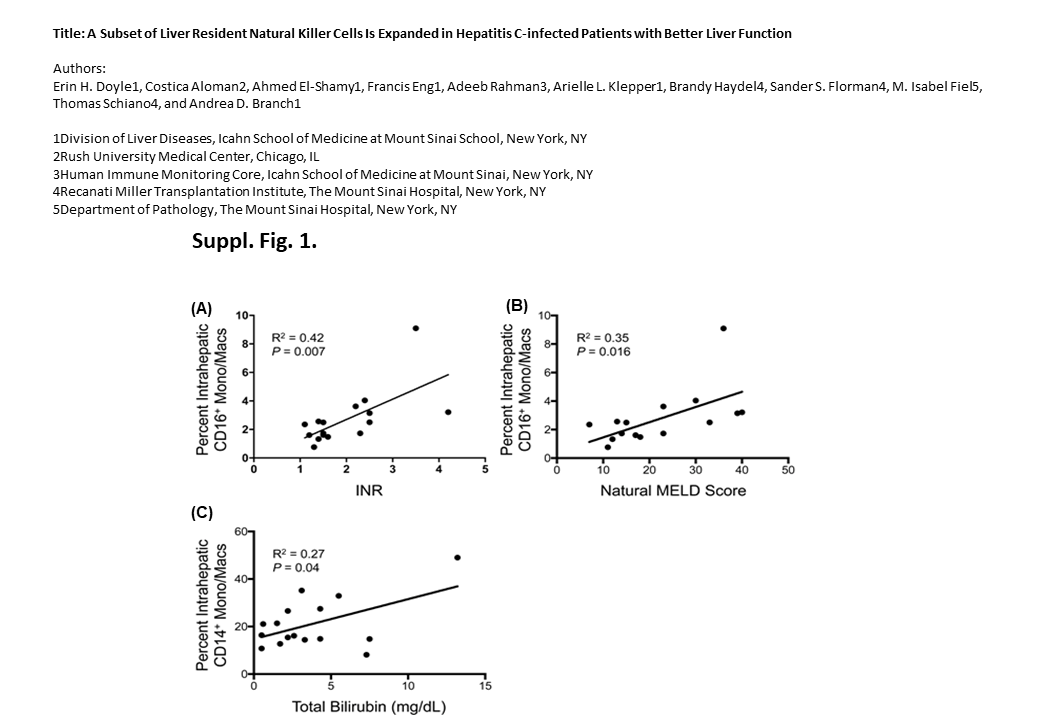

Supplement: Supplementary file 2 — Supplementary Figure S1. [file 41598_2020_80819_MOESM2_ESM.tif]
